# Comparison of the Distal Gut Microbiota from People and Animals in Africa

**DOI:** 10.1371/journal.pone.0054783

**Published:** 2013-01-23

**Authors:** Richard J. Ellis, Kenneth D. Bruce, Claire Jenkins, J. Russell Stothard, Lilly Ajarova, Lawrence Mugisha, Mark E. Viney

**Affiliations:** 1 Specialist Scientific Support Department, Animal Health and Veterinary Laboratories Agency, Addlestone, Surrey, United Kingdom; 2 Institute of Pharmaceutical Science, King’s College London, London, United Kingdom; 3 Laboratory of Gastrointestinal Pathogens, Health Protection Agency, London, United Kingdom; 4 Disease Control Strategy Group, Liverpool School of Tropical Medicine, Liverpool, United Kingdom; 5 Chimpanzee Sanctuary and Wildlife Conservation Trust (CSWCT), Entebbe, Uganda; 6 College of Veterinary Medicine (Animal Resources and Biosecurity), Makerere University, Kampala, Uganda; 7 Conservation & Ecosystem Health Alliance (CEHA), Kampala, Uganda; 8 School of Biological Sciences, University of Bristol, Bristol, United Kingdom; Institute for Genome Sciences, University of Maryland School of Medicine, United States of America

## Abstract

The gut microbiota plays a key role in the maintenance of healthy gut function as well as many other aspects of health. High-throughput sequence analyses have revealed the composition of the gut microbiota, showing that there is a core signature to the human gut microbiota, as well as variation in its composition between people. The gut microbiota of animals is also being investigated. We are interested in the relationship between bacterial taxa of the human gut microbiota and those in the gut microbiota of domestic and semi-wild animals. While it is clear that some human gut bacterial pathogens come from animals (showing that human – animal transmission occurs), the extent to which the usually non-pathogenic commensal taxa are shared between humans and animals has not been explored. To investigate this we compared the distal gut microbiota of humans, cattle and semi-captive chimpanzees in communities that are geographically sympatric in Uganda. The gut microbiotas of these three host species could be distinguished by the different proportions of bacterial taxa present. We defined multiple operational taxonomic units (OTUs) by sequence similarity and found evidence that some OTUs were common between human, cattle and chimpanzees, with the largest number of shared OTUs occurring between chimpanzees and humans, as might be expected with their close physiological similarity. These results show the potential for the sharing of usually commensal bacterial taxa between humans and other animals. This suggests that further investigation of this phenomenon is needed to fully understand how it drives the composition of human and animal gut microbiotas.

## Introduction

It has become increasingly clear that the gut microbiota plays a key role in the maintenance of normal gut function, the digestion of food and much wider aspects of human health, though much remains to be discovered [1]–[6]. Analysis of the gut microbiota is rapidly increasing through the use of next generation sequencing. The gut microbiota has, however, long been recognized to contain a complex mix of mostly (in terms of biomass) bacterial taxa, the resolution of which is now rapidly increasing. Individuals begin to be colonised with microbes from birth [7], with maternal and childhood events considered to have life-long effects [8], [9]. During the first months of life the composition of individual microbiotas vary, but this stabilizes to a mix of major bacterial phyla (Firmicutes, Bacteroidetes *etc*. [10], [11]) as the child is weaned and by the age of three, which then persists throughout life [12]–[15]. However, the gut microbiota does undergo further maturation (in terms of the constituent species within each of the major phyla) in adulthood and alters again in old age [16]. Notwithstanding this general pattern, there is a large degree of inter-individual variation in microbiotas, such that individuals have their own “fingerprint” of microbial taxa [10]. There is both an environmental (*e.g.* microbial exposure, diet, infection effects [12], [13], [17]–[20]) and a genetic component [1], [14], [21], [22] controlling the composition of the microbiota. Seemingly subtle fluctuations in the intestinal ecosystem may be important in explaining differential susceptibility to pathological episodes [19]. Recent studies have examined the community composition of the gut microbiota in people of different ages [23], [24]; in infection e.g. *Clostridium difficile* [25] and in inflammatory bowel disease [26]–[28]. Moreover, studies have started to investigate how the gut microbiota differs between different human populations, for example between children in Italy and Burkina Faso [17], or on a larger scale, comparing multiple age ranges of human populations in Venezuela, Malawi and US [15]. In the former study, African children were found to have a higher relative abundance of bacteria belonging to the Bacteroidetes group and a lower relative abundance of taxa from the Firmicutes group, compared to their Italian counterparts [17]. This difference has been interpreted to be due to possible effects of geography and/or diet. While these are intriguing findings, there seem to be substantial between-individual differences in the microbiotas (but which can be subject to short-term perturbation).

In addition to studies of the human gut microbiota, other work has compared the microbiota from different species of non-human primates [29], [30], finding that variation in the composition of distal gut microbiota can often be related to evolutionary distances among these host species [29], [30]. Other recent studies have also suggested that diet differences among mammalian taxa drive the composition of the microbiota, and thus its functioning [31]. There have also been studies of domesticated animals, which have found interesting differences between the faecal microbiota of beef and of dairy cattle [32]. Although it is likely to be an area of considerable importance [15], few studies have focused on whether there are any similarities between the bacterial taxa present in humans and either domesticated or semi-wild animals. A key article has shown that host diet and phylogenetic relatedness of the hosts both influence the bacterial community structure and that human gut microbiota is similar in composition to that of other omnivorous primates [33]. However, beyond these gross-level differences the presence of identical operational taxonomic units (OTUs) in different hosts has not been examined. Some studies have described differences between the components of the microbiota in faecal samples from a variety of animals and humans for the purpose of microbial source tracking [34], [35], but these data were not examined in depth to determine the extent to which taxa may be shared among host species. It is clear that some human gut bacterial pathogens come from animals (i.e. they are zoonotic, showing the potential for animal to human transmission of bacterial taxa), but the extent to which the usually non-pathogenic commensal taxa are shared with and/or derived from animals species has not been explored.

In this study, we sought to further examine the composition of distal gut microbiotas in order to investigate the extent of any commonality at the level of individual OTUs between humans and domesticated or semi-wild animals. Using next-generation sequencing of 16S ribosomal RNA gene amplicons, the microbial communities of faecal samples from humans, domestic cattle and semi-captive chimpanzees from communities that are geographically sympatric were compared. Here we demonstrate that a considerable proportion of OTUs are shared between all three host species, while there are also phylum- and family-level differences in the composition of these microbiotas.

## Materials and Methods

### Sampling and Ethics Statement

Faecal samples were collected from people from the village of Bugoto, on the shoreline of Lake Victoria, Mayuge District, Uganda. These consisted of five mother and child (≤5 years of age) pairs and 6 adult males (age 30–45 years old) whose relationships to the mother and child pairs were unknown. These participants form part of an ongoing longitudinal cohort investigation monitoring the dynamics of intestinal schistosomiasis and malaria in this lakeshore community. Stool samples were collected after obtaining written informed consent with the exception that each mother provided assent on behalf of her child. After collection, faecal specimens were anonymized whilst keeping both mother and child pairs and gender information intact. The study was granted ethical approval from the London School of Hygiene and Tropical Medicine (Ref 5538.09) and the Ugandan National Council of Science and Technology (HS 748).

For local comparisons, further faecal samples were obtained by passive collection from five grazing cattle from the shoreline pasture of Bugoto village, immediately adjacent to where the human field clinics were taking place. Samples were collected non-invasively from normal village livestock so no specific ethical clearance was deemed necessary for these cattle samples.

For comparison with non-human primates in this Lake Victoria setting, faecal samples were also collected from Ngamba Island Chimpanzee Sanctuary (Mukono District), an island in Lake Victoria containing a reserve for wild-born, semi-captive, chimpanzees. These animals have been rescued from poachers at a young age (2–5 years) and placed under long term care at the sanctuary in a semi-captive environment which maintains a high level of welfare and husbandry practices that mimic the natural behaviours of the species [36]. The sanctuary is the member of the Pan African Sanctuaries Alliance (PASA) which promotes and monitors high ethical conducts of sanctuaries. Ngamba Island Chimpanzee Sanctuary exhibits high level of professionalism and care of the rescued chimpanzees and has thus been recognized with certification from the Global Federation of Animal Sanctuaries (GFAS) for standards of excellence in humane and responsible care for animals and guidelines defining ethical and legal sanctuary. Faecal samples from 6 adult (>12 years) male chimpanzees and 5 adult male staff of the sanctuary were obtained following approval from the sanctuary management as part of routine health monitoring and research approval from the Uganda Wildlife Authority (UWA). All stool samples were collected non-invasively from chimpanzees from their sleeping enclosure facility and from staff (caregivers) during an annual intestinal schistosomiasis and malaria spot-check screen. All specimens were collected as soon as possible after defecation and always within 1 hour. All faecal specimens were processed within 2–3 hours of collection.

From the specimens collected, each faecal sample was treated identically; by filtration through a 212 µm metal sieve mesh from which a 0.5 g pellet was obtained under aerobic conditions. This was then placed in a 15 ml tube to which approximately 7 ml of RNAlater was added. The faecal pellet was homogenized using an electric vortex machine and all samples were stored at room temperature prior to transfer to the UK for DNA extraction. Importation of samples to the UK was licensed by Department for Environment Food and Rural Affairs (PATH/125/2011/2).

### DNA Extraction

Faecal samples were mechanically disrupted with a pre-treatment step to aid the extraction of nucleic acids. Briefly, 200 µl of each faecal sample was added to MagNa-Lyser Green Beads tubes (Roche Diagnostics Ltd, UK) containing 900 µl L6 lysis buffer (Severn Biotech, UK) and 20 µl isoamyl alcohol. The tubes were shaken for 1 min in the MagNa-Lyser Bead-Beater instrument (Roche Diagnostics Ltd, UK) at 3,000 rpm then centrifuged for 30 sec at 12,000 *g*. 250 µl of the supernatant was transferred into a sterile 2 ml screw cap tube containing 250 µl PBS. Each sample was vortexed to ensure even distribution and loaded onto the QIAsymphony automated extraction platform (Qiagen, UK) and DNA was extracted following the manufacturer’s instructions. During the extraction process, samples are lysed under denaturing conditions in the presence of proteinase K, and the DNA binds to the silica surface of magnetic particles; contaminants were removed by washing, and pure DNA was eluted in modified TE buffer (Qiagen, UK). Each batch of extractions was performed with one tube containing water, as a negative control.

### Amplification and High-throughput Sequencing of 16S rRNA Gene Regions

Aliquots of extracted DNA were amplified with universal primers for the V4 and V5 regions of the 16S rRNA gene. The primers U515F (5′-GTGYCAGCMGCCGCGGTA) and U927R (5′-CCCGYCAATTCMTTTRAGT) were designed to permit amplification of both bacterial and archaeal ribosomal gene regions, whilst providing the best possible taxonomic resolution based on published information [37], [38]. Forward fusion primers consisted of the GS FLX Titanium primer A and the library key (5′-CCATCTCATCCCTGCGTGTCTCCGACTCAG) together with one of a suite of eight 10 base multiplex identifiers (MIDs 1–8) (Roche Diagnostics Ltd, UK). There were at least two base differences between each pair of MIDs thus reducing the possibility of misidentification at the demultiplexing stage. Reverse fusion primers included the GS FLX Titanium primer B and the library key (5′-CCTATCCCCTGTGTGCCTTGGCAGTCTCAG). Amplification was performed with FastStart HiFi Polymerase (Roche Diagnostics Ltd, UK) using the following cycling conditions: 94°C for 3 min; 30 cycles of 94°C for 30 s, 55°C for 45 s, 72°C for 1 min; followed by 72°C for 8 min. After initial failure to produce sufficient material for further analysis for some sample extracts after 25 PCR cycles we increased the number of PCR cycles to 30 for all samples. Amplicons were purified using Ampure XP magnetic beads (Beckman Coulter) and the concentration of each sample was measured using the fluorescence-based Picogreen assay (Invitrogen). Concentrations were normalized before pooling samples in four batches of 8, each of which would be subsequently identified by its unique MID. Each of the four pools were then subjected to unidirectional sequencing from the forward primer in separate picotitre plate regions on the GS FLX Titanium platform according to the manufacturer’s instructions (Roche Diagnostics).

### Data Processing and Analysis

The total data set consisted of 119,445 reads that passed quality filtering and were over 50 bases in length and these have been deposited at the Sequence Read Archive at NCBI (accession numbers to follow). The data were processed using the Quantitative Insights Into Microbial Ecology software package (QIIME v1.3.0) [39] as implemented in Biolinux 6 [40]. Initially, the Ampliconnoise pipeline [41] was used to split the dataset into separate files for each sample according to the MID adaptors used, and then to remove pyrosequencing errors, PCR errors and chimeric sequences. Only sequences over 400 bases in length were retained for further analysis. The denoised data were used to produce a representative set of OTUs (97% similarity cut-off), which were then aligned and clustered using uclust [42] and PyNast [43]. Taxonomy was assigned according to the RDP classifier [37]. The relative abundance of taxa at multiple levels of resolution (phylum, order, family, etc) was then determined for each sample. Jackknifed beta-diversity of the data set was calculated using the unweighted UniFrac metric [44] as implemented in QIIME using a re-sampling size of 250. Principal coordinates plots of the UniFrac distance matrices were then generated to investigate the relationships between microbiotas in each of the samples. The G-test of independence was used to determine if the most abundant OTUs were non-randomly distributed between different host species. The false discovery rate approach was used to correct for multiple testing errors.

Phylogenetic trees of specific OTUs were produced by searching for nearest neighbours for all sequences in a sample using the standard RDP Seq Match tool [45]. Sequences were downloaded directly from RDP and trimmed to uniform sequence length before aligning with representative OTU sequences from our dataset using clustalX [46]. Neighbour-joining trees were constructed and visualized with MEGA4 [47].

## Results

Following all denoising and filtering steps in QIIME, a total of 45,370 (mean 2,160, n = 21) sequences from humans, 16,043 (mean 2,674, n = 6) sequences from chimpanzees and 11,354 (mean 2,271, n = 5) sequences from cattle were used in the final analysis. The ability of our primers to amplify archaeal sequences was demonstrated by the presence of 146 (0.02% overall abundance) archaeal ribosomal gene sequences in the dataset (all within Euryarchaeota: *Methanobrevibacter*, *Methanocorpusculum* and 5 other OTUs that were not classified beyond the phylum level) from cattle, chimpanzee and human samples. However, 107 (73%) of these archaeal sequences were from chimpanzee samples, but due to their low overall abundance they will not be considered in detail here.

**Figure 1:**
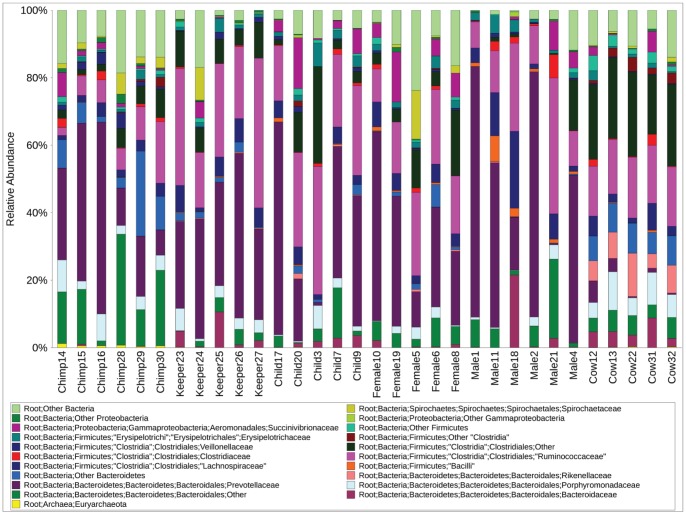
The relative abundance of dominant bacterial families among gut microbiotas from humans, cattle and chimpanzees. Classification is based on Ribosomal Database Project taxonomy.

The relative proportion of taxa among bacterial families from each of these samples varied considerably (Figure 1). However, among all host species, taxa within the Bacteroidetes and Firmicutes phyla were dominant, but their relative proportions differed (Table 1). Samples from chimpanzees had a significantly lower proportion of Firmicutes taxa (*P<*0.0025*)* compared with humans and cattle (Table 1). Comparison of standard deviations of the proportions of these dominant phyla showed that there was less inter-individual variation among the cattle samples compared with the samples from humans or chimpanzees, and this is also reflected at the family-level analysis as also seen in Figure 1. Among the human samples there were substantial differences in the relative proportions of these phyla, ranging between 14–83% Bacteroidetes and 15–77% Firmicutes.

**Table 1:** Relative abundance of the Bacteroidetes and Firmicutes phyla in each host species.

	Mean relative abundance (SD) [Minimum – Maximum]	
	Bacteroidetes (%)	Firmicutes (%)
**Human**	47.4 (3.8) [14.4–83.3]	41.0 (2.8) [15.4–76.9]
**Cattle**	36.0 (0.2) [32.9–43.0]	51.9 (0.0) [50.1–53.4]
**Chimpanzee**	59.7 (1.0) [45.5–73.2]	21.4 (1.2) [12.8–38.4]

**Figure 2:**
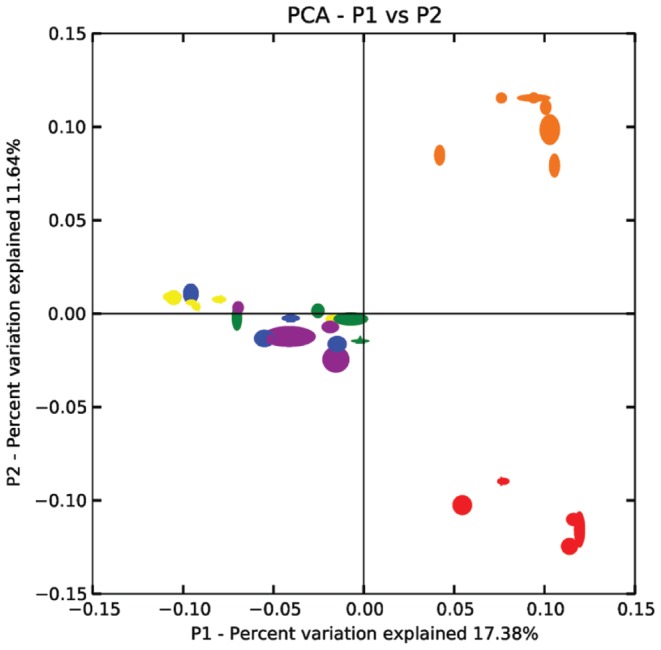
Principal Coordinates Analysis of the dissimilarity of microbial communities of the 32 samples. Dissimilarities were calculated using the UniFrac metric based on a re-sampling size of 250 sequences. Individual samples are indicated by small symbols and each halo indicates the variability calculated from Jackknife re-sampling. Different symbol colours and shapes represent different sample categories: (green ▴) female, (blue ▪) child, (yellow ◂) male, (red ▸) cattle, (orange •) chimpanzee and (lilac ▾) keeper.

Of the 72,767 reads used in the analysis, a total of 2,218 OTUs were defined with a sequence similarity of greater than 97%. A plot of the first two principal coordinates of the unweighted UniFrac distance matrices showed that there is minimal variation within host species compared to the differences among microbiotas from the three host species (Figure 2). Samples from humans were primarily separated from non-human samples by PC1, but PC2 separated all three groups. Further, there were no discernible differences between the 4 sub-sources of human samples (i.e. male staff from Ngamba, Children, Females or Males from Bugoto).

**Figure 3:**
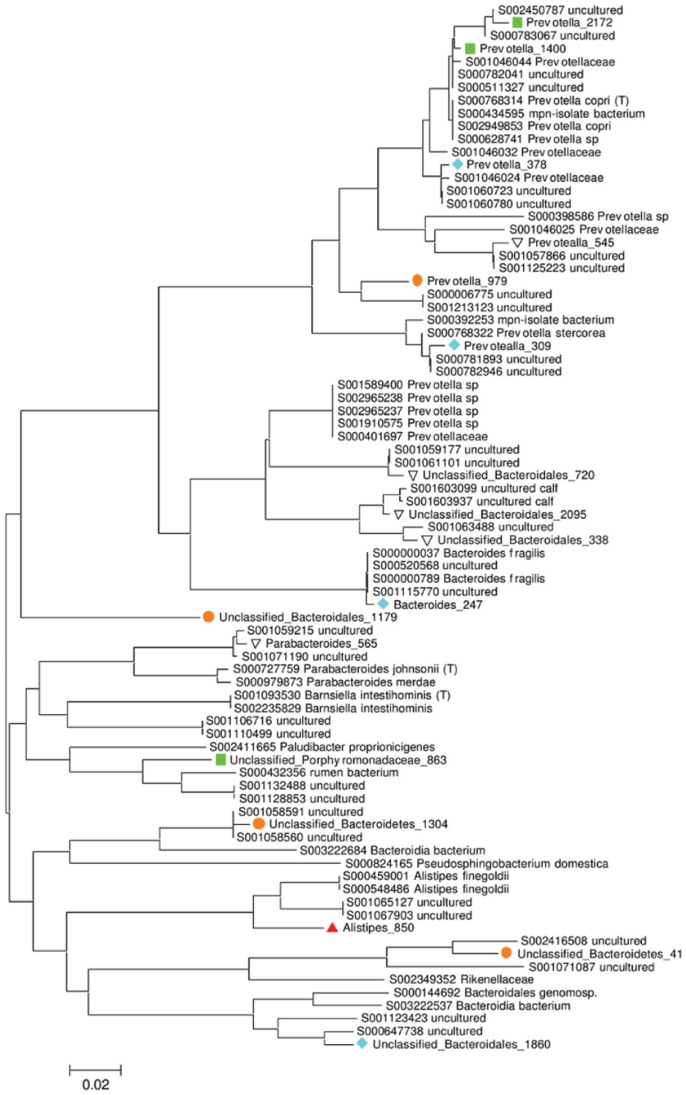
Figure **3.** **Phylogenetic tree showing the most abundant OTUs within the Bacteroidetes phylum.** Phylogeny is based on 400 bp alignment of the V4–V5 of the 16S rRNA gene, together with the closest matches from the Ribosomal Database Project. Together these 17 OTUs comprise 37.7% of the total sequences and 75.3% of those within the Bacteroidetes phylum. RDP sequences are labelled with their “S” accession number and taxonomic assignment. Coloured symbols indicate the distribution of OTUs (defined as 97% sequence similarity) that are shared between hosts; (green ▪) humans, cattle and chimpanzees, (blue ♦) humans and chimpanzees, (orange •) chimpanzees only, and (red ▴) cattle only. Each OTU name consists of its taxonomic assignment and a unique numerical identifier. Scale bars indicate the number of base substitutions per site.

Of the 2,218 OTUs, none were present in all 32 samples, but 880 OTUs were singletons. In the detailed analysis described below each OTU has been named according to the taxonomic level to which it can be assigned with greater than 80% confidence, and given a unique numerical OTU identifier. Those taxa not assigned to genus level are prefixed with ‘Unclassified’. A total of 423 (19.1% of all OTUs identified; 31.6% of non-singleton OTUs) OTUs were recovered from more than one host species. We further investigated how the most abundant OTUs (each >0.5% abundance) were distributed among the host species (Table 2). Twenty-eight OTUs were in this category, but accounted for 53.6% of the total number of sequences in the dataset and of these 16 (57%) OTUs were shared between multiple host species. Figure 3 shows the phylogenetic tree for 17 OTUs from the Bacteroidetes that are above 0.5% total abundance, illustrating that two closely-related taxa, *Prevotella*_1400 and *Prevotella*_2172, are shared between all three host species. Table 2 shows the distribution and abundance of these taxa in more detail, showing that *Prevotella*_1400 is the single most abundant OTU, accounting for 19.2% of the entire dataset. Further, this OTU is present in 27 of the 32 samples (representing all three host species), but is more common in humans and chimpanzees than it is in cattle. *Prevotella*_2172 is also present, though relatively scarce, in all three host species. Two further OTUs assigned to the *Prevotella* genus (*Prevotella_*309 and *Prevotella_*378) together with *Bacteroides*_247 are common in humans and chimpanzees but absent from cattle. There were 5 further Bacteroidetes OTUs that were only recovered from chimpanzees, but only one of these (*Prevotella*_979) could be classified below the order level. Only 2 OTUs within the abundant Bacteroidetes shown in Table2 were specific to cattle; Unclassified_*Porphyromonadaceae*_863 and *Alistipes*_850.

**Table 2:** Distribution of the most abundant OTUs (>0.5% of total abundance) in humans, chimpanzees and cattle.

Phylum	OTU	Taxonomy	Human (n = 21)		Chimpanzee (n = 6)		Cattle (n = 5)		Significance^a^
		Confidence	Samples	Sequences	Samples	Sequences	Samples	Sequences	
Bacteroidetes	Unclassifed_Bacteroidetes_1304	1.00	0		5	660	0		0.0019
	Unclassifed_Bacteroidetes_41	0.98	0		6	401	0		0.0002
	Unclassifed_Bacteroidales_338	0.87	5	669	0		0		−
	Unclassifed_Bacteroidales_720	0.89	11	643	0		0		−
	Unclassifed_Bacteroidales_1179	0.84	0		6	1195	0		0.0002
	Unclassifed_Bacteroidales_1860	0.85	1	3	6	1697	0		0.0006
	Unclassifed_Bacteroidales_2095	0.94	15	901	2	2	0		−
	*Bacteroides*_247	1.00	17	854	2	7	0		0.0284
	Unclassifed_Porphyromonadaceae_863	0.93	0		0		5	575	0.0004
	*Parabacteroides*_565	0.81	7	418	0		0		−
	*Prevotella*_309	0.98	17	2350	5	39	0		0.0428
	*Prevotella*_378	0.99	11	442	6	232	0		0.0306
	*Prevotella*_979	0.85	0		6	486	0		0.0005
	*Prevotella*_545	0.99	2	796	0		0		−
	*Prevotella*_1400	0.99	19	12723	6	1236	2	4	−
	*Prevotella*_2172	1.00	12	406	3	14	2	37	−
	*Alistipes*_850	0.99	0		0		5	667	0.0003
Firmicutes	Unclassifed_Clostridiales_742	0.89	4	258	5	132	0		−
	Unclassifed_Clostridiales_1602	0.97	17	349	6	41	5	84	−
	*Roseburia*_226	0.80	17	655	6	65	3	8	−
	Unclassifed_Ruminococcaceae_442	0.98	20	619	5	59	5	629	−
	*Faecalibacterium*_748	0.98	21	3715	6	141	0		0.0004
	*Ruminococcus*_1290	0.99	14	647	6	21	5	26	−
	*Sporobacter*_4	0.91	17	727	4	264	2	20	−
	*Catenibacterium*_1451	1.00	18	487	0		0		0.0004
Proteobacteria	*Succinivibrio*_100	1.00	14	890	6	246	0		0.0283
	*Ruminobacter*_1102	0.96	6	559	4	45	3	51	−
Spirochaetes	*Treponema*_358	0.97	3	785	0		0		−

a Probability that the OTU is non-randomly distributed between humans, chimpanzees and cattle, calculated using the G test of independence after correction for the ‘False Discovery Rate’. All probabilities of <0.05 are shown. Where no values are shown there is not considered to be a significant non-random distribution.

**Figure 4:**
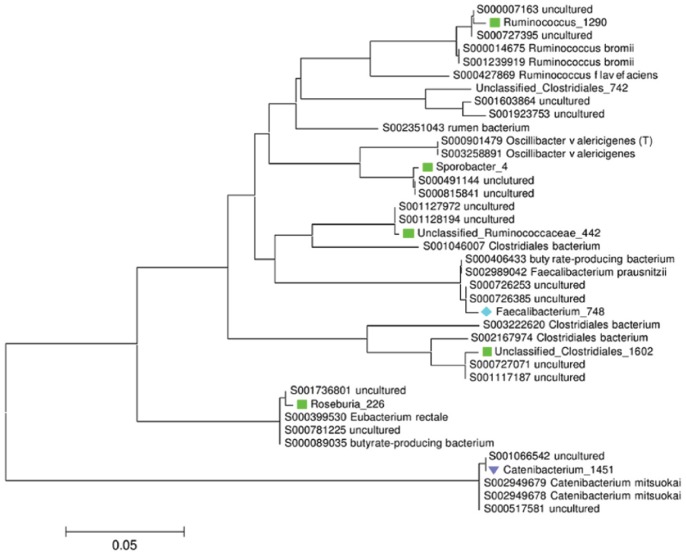
Figure **4.** **Phylogenetic tree showing the most abundant OTUs within the Firmicutes phylum.** Phylogeny is based on 400 bp alignment of the V4–V5 of the 16S rRNA gene showing, together with the closest matches from the Ribosomal Database Project. Together these 8 OTUs comprise 12.3% of the total sequences and 33.2% of those within the Firmicutes phylum. RDP sequences are labelled with their “S” accession number and taxonomic assignment. Coloured symbols indicate the distribution of OTUs (defined as 97% sequence similarity) that are shared between hosts; (green ▪) humans, cattle and chimpanzees, (blue ♦) humans and chimpanzees, (lilac ▾) humans only. Each OTU name consists of its taxonomic assignment and a unique numerical identifier. Scale bars indicate the number of base substitutions per site.

The phylogenetic tree for the abundant Firmicutes taxa is shown in Figure 4, with corresponding numerical data in Table 2. It is clear that the dominant Firmicutes taxa in the human samples consisted of representatives of several genera (*Faecalibacterium*, *Sporobacter*, *Roseburia*, and *Ruminococcus*), all of which were either shared with chimpanzees, cattle, or both. Additional unclassified Ruminococcaceae and Clostridiales OTUs were also found in at least 2 host species. However, *Catenibacterium*_1451 was restricted to humans. Individual Firmicutes OTUs generally have a lower relative abundance in samples, but *Faecalibacterium*_748 was the only OTU present in all 21 human samples.

Table 2 also shows that a few OTUs from other phyla had an overall abundance of >0.5%. These were *Succinovibrio*_100, *Ruminobacter*_1102 both from the Proteobacteria and *Treponema*_358 from the Spirochaetes. The latter was found in only 3 human samples (but at a high relative abundance), whilst both Proteobacteria OTUs were recovered from at least 2 host species, but the absence of *Succinovibrio*_100 from cattle was notable (Table 2).

**Figure 5:**
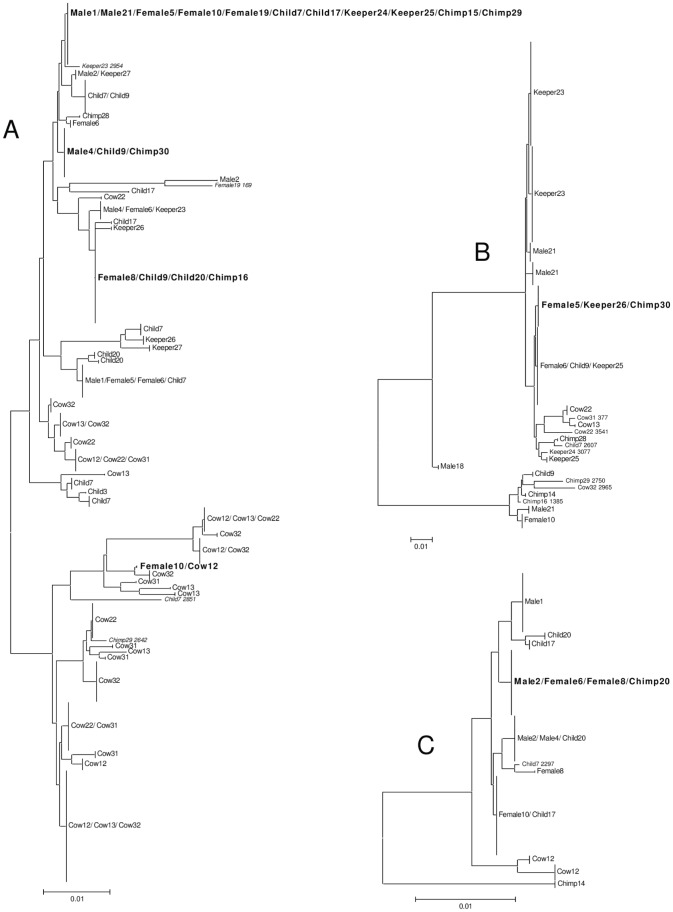
Figure **5.** **Phylogenetic trees of 3 selected OTUs.** (A) Unclassified_Ruminococcaceae_442, (B) *Ruminococcus*_1290 OTU and (C) *Prevotella*_2172. Branches are labelled with the sample from which the sequence originated (labelled as in Figure 1). Branch labels in bold are where an identical sequence was obtained from samples from more than one host species. Scale bars indicate the number of base substitutions per site.

In addition to the usual analysis of gut microbiota in terms of OTU composition we also examined the phylogeny and provenance of individual sequences with three of the widely distributed (i.e. in all three host species) OTUs; Unclassified_Ruminococcaceae_442, *Ruminococcus*_1290 and *Prevotella*_2172. Figure 5 shows the phylogenetic trees for each of these OTUs. Each OTU consisted of multiple sequences but this is consistent with natural molecular variation within bacterial taxa. In general, any single sequence within these OTUs was found in one individual sample. It should be noted that for all these three OTUs, the sequences from cow samples were generally distinct from those of chimpanzees and humans. However, for all of these three OTUs there were individual sequences (*i.e.* with 100% similarity) that were found in multiple samples, and for six cases from more than one host species. For example, identical sequences from all three OTUs (Unclassified_Ruminococcaceae_442, *Ruminococcus*_1290 and *Prevotella*_2172) were found in samples from both humans and chimpanzees. In addition, there is one example of the same Unclassified_Ruminococcaceae_442 sequence being found in both a human and cow sample.

## Discussion

This work has compared the composition of the gut microbiota from humans, domestic cattle and semi-captive chimpanzees all originating from environments that fringe the Lake Victoria shoreline. Many recent studies have examined the gut microbiota of these host species, describing the relative abundances of different taxonomic groups. The principal aim of this study was to investigate the extent to which individual bacterial OTUs are shared within and between the gut microbiotas of different host species set within geographical sympatry where local opportunities for transmission and colonisation are perhaps greatest. However, to provide an overview of these microbiotas and to place this work in the context of other recent studies, we also include higher taxonomic-level analyses, which showed that the same two phyla (Bacteroidetes and Firmicutes) were the most abundant within the microbiotas of all three host species, but that the relative abundance of each differed between them. There was also substantial variation in the abundance of these phyla among the human samples. Notably, our results differ from previous work comparing human microbiotas between continents which found that Bacteroidetes dominated in Burkinabes, but Firmicutes dominated in Europeans [17]. However, even within the relatively small number of samples that we examined from Uganda, there were some individual samples that were dominated by Bacteroidetes whilst others were dominated by Firmicutes. Another finding from the African-European comparison was that *Prevotella* was the most common genus in the Bacteroidetes population from African samples [17], which was also found in our study. Conversely, *Xylanibacter* were not dominant in the human samples from our study although they were in the samples from West Africa [17]. This disparity may be due to dietary differences between these different geographical locations. Likewise, the phylum-level composition of the cattle and chimpanzee microbiotas from this study were similar to those that have been previously published [29], [48]. Taken together, the similarities between our results and those of other studies indicate that, at this phylum-level of analysis, the composition of gut microbiota is remarkably stable within individual host species despite differences in geography and diet, or even differences in sample handling, processing procedures and sequencing strategies.

Comparison of the microbiota composition at higher taxonomic levels (e.g. phylum or order) is useful for determining gross differences among samples. However, we wanted to determine whether the same OTUs were shared among host species, and to that we end we interrogated our data set in greater detail to determine the degree to which each OTU was distributed among samples. The 97% similarity cut-off that was used when assigning OTUs was selected to approximate the 16S rRNA gene sequence variation seen within bacterial species. Clearly such a cut-off has its limitations, because 16S rRNA gene sequence variation between some described genera (e.g. *Escherichia* and *Shigella*) is less than 97%, whilst the variation between 16S rRNA gene copies within individual genomes can also exceed 97% [49]. Furthermore, the relevance of the species concept for bacteria is currently receiving considerable attention [50]. However, given these limitations, our results provide evidence that a considerable proportion (19.1%) of bacterial OTUs (as classified by the 97% cut-off) may be shared between different host species. Clearly greater characterisation of bacterial genomes would be required to determine the extent to which identical bacteria may be shared between different host species. Within both the Bacteroidetes and Firmicutes phyla, the microbiotas from the human samples had OTUs in common with samples from both animal species. While there were many OTUs shared between samples from humans and chimpanzees but not cattle, there were no OTUs shared between cattle and only one other host species. The greater similarity between the microbiota of humans and chimpanzees, than between that of primates and cattle, could be related to the relative phylogenetic differences between these host species [29], [30], [33], as well as differences in diet which has recently been shown to be important in driving the function and therefore the composition of the gut microbiota [31]. Notwithstanding, our key observation is that the same OTUs (with >97% similarity at the sequence level) can be found in samples from multiple host species. Furthermore, by investigating all sequence reads assigned to select OTUs in detail, we have also shown that identical 400 base sequences can be recovered from multiple host species. The realistic threat of zoonotic pathogens being transferred between animal hosts and human populations is already well known, but our data suggest that this phenomenon may also apply to commensal, non-pathogenic bacterial taxa.

How these bacterial OTUs come to occur in multiple host species remains to be elucidated, though in principle this could occur either by direct host – host contact, or indirectly, for example *via* environmental contamination which could be enhanced on this lakeshore setting where faecal material can be easily dispersed in the water margins. Alternatively, the environment could be a source of bacteria for all host species without a specific need for faecal-oral contamination. We obtained samples from geographically sympatric communities and it is clear that there is the potential for contact for some humans with either the chimpanzees or the cattle included in this study, but direct contact alone cannot account for the occurrence of identical 16S rRNA gene sequences in multiple samples. For example, whilst the occurrence of identical sequences in samples from keeper and semi-captive chimpanzee samples could be explained by direct transmission, this cannot explain their occurrence in samples from humans in a village over 100 km away although set within a similar lakeshore setting.

A larger proportion of the Firmicutes OTUs were shared among human, chimpanzee and cattle samples, compared to the Bacteroidetes OTUs. Why Firmicutes OTUs are apparently preferentially shared among host species also requires further investigation. Some Firmicutes species form spores that would readily facilitate transfer via the environment, but others such as *Faecalibacterium* (which were common in the human and chimpanzee samples) do not. Alternatively, the distribution of certain taxa across multiple host species could be due to the fact that they bestow their host with some essential function that cannot be provided by other taxa.

Our analysis shows that there are some bacterial OTUs that are specific to particular host species, whilst others appear to be more ubiquitous. Previous work has shown that compositional differences in gut microbiota can be related to age, diet, health etc [15], [33], but we have demonstrated that despite such differences, a substantial proportion of bacterial OTUs (defined as >97% sequence similarity) recur both within and between host species. Moreover, identical 400 base sequences within OTUs can be recovered from multiple host species, reinforcing the observation that these OTUs are indeed shared, rather than being divergent variants of some ancestral taxa. However, our data cannot rule out the possibility that OTUs in different host species carry host-specific adaptations; such analysis is likely to require whole genome sequencing of multiple isolates from different hosts. We have shown that different taxa have distinct patterns of distribution among host species, suggesting that they each perform distinct roles in their host gut, and their presence in a particular sample will be a result of the bacterial function and the host requirement for that function [15], [51]. Recent work has shown that some bacterial species are responsible for starch degradation [51] whilst others are involved in vitamin biosynthesis [15] in the host gut. Clearly a greater understanding of the function of individual bacterial species is required if we are to fully understand the compositional variation in gut microbiota. To this end, whole genome comparisons of key bacterial OTUs from various host species and populations within those hosts will provide further insight.
